# Simultaneous determination of cytochrome P450 1A, 2A and 3A activities in porcine liver microsomes

**DOI:** 10.2478/v10102-012-0024-3

**Published:** 2012-09

**Authors:** Monika Johansson, Jana Tomankova, Shengjie Li, Galia Zamaratskaia

**Affiliations:** 1 Department of Food Science, BioCenter, Swedish University of Agricultural Sciences, Uppsala, Sweden; 2 University of Veterinary and Pharmaceutical Science Brno, Faculty of Hygiene and Ecology, Department of Meat Hygiene and Technology, Brno, Czech Republic

**Keywords:** hepatic microsomes, cytochrome P450, multiple probe substrates, HPLC

## Abstract

The aim of this study was to develop a robust method for the simultaneous determination of the activities of three porcine CYP450 enzymes in hepatic microsomes. A cocktail consisting of three selective CYP450 probe substrates, 7-ethoxyresorufin (CYP1A), coumarin (CYP2A) and 7-benzyloxy-4-trifluoromethylcoumarin (BFC; CYP3A), was incubated with porcine liver microsomes. The presence of 7-ethoxyresorufin appears to significantly influence the kinetics of coumarin hydroxylation and BFC *O*-debenzylation. These results indicate that the use of 7-ethoxyresorufin in substrate cocktails together with coumarin and BFC should be avoided.

## Introduction

Cytochrome P450 (CYP450) is a superfamily of enzymes involved in the metabolism of a large number of endogenous and exogenous compounds including drugs and environmental pollutants (Anzenbacher & Anzenbacherová, 2001). The catalytic activity of CYP450 enzymes can be affected by various factors, including genetic background, age, sex and diet (Glue & Clement, 1999). An assessment of the CYP450 activity is important in toxicologic and pharmaceutical studies. A variety of assays were developed to assess the activity of specific CYP450 isoforms using variable probe substrates. Generally, for activity determination, a known substrate for a specific CYP enzyme is incubated with microsomes in the presence of NADPH and the concentration of the formed substrate metabolite is measured. The main limitations of such assays are a low speed of quantification and high amount of an enzyme source (Hickman *et al.*, 1998). Thus, the development of a method to simultaneously assess several isozymes is beneficial because it can drastically reduce the time and cost of analysis. Recently, a number of methods in which a cocktail of CYP substrates was used in a single microsomal incubation were developed (Jurica *et al.*, 2009; Alden *et al.*, 2010; Otten *et al.*, 2011).

Hepatic microsomes, a subcellular fraction containing the CYP450 enzymes, are widely used to evaluate metabolic biotransformation of various compounds and to identify enzyme or enzymes responsible for these biotransformations.

The aim of this study was to develop a new, robust method for the simultaneous estimation of the activities of three porcine CYP450 enzymes in hepatic microsomes. For this purpose, we used a cocktail of known CYP450 probe substrates, ethoxyresorufin (CYP1A), coumarin (CYP2A) and 7-benzyloxy-4-trifluoromethylcoumarin (BFC; CYP3A).

## Methods

### Animals

Entire male pigs of a crossbred (Swedish Yorkshire dams × Landrace sires) raised at the experimental station Funbo-Lövsta at the Swedish University of Agricultural Sciences, were included in the study. The pigs were fed the same commercial diet according to the standard feeding regimen for finishing pigs in Sweden (restricted, 12 MJ ME per kg, digestible CP 13%) and were slaughtered at an average of 115 kg of live weight. Liver samples from all animals were taken at slaughter, frozen in liquid nitrogen and stored at –80°C until use.

### Microsome preparation

Microsomes were prepared from porcine liver by a calcium aggregation method using TRIS-EDTA homogenization buffer (Rasmussen *et al.*, 2011). Protein concentrations in the microsomes were measured with a commercially available kit (Bio-Rad Laboratories Inc., Hercules, CA, USA) according to the manufacturer's instructions. Then the microsomes were stored at −80°C until use. In each experimental set, two pools of liver microsomes from 4–5 pigs were used. Total protein concentration was measured with Bio-Rad protein assay kit (Bio-Rad laboratories Inc., Hercules, CA, USA) according to the manufacturer's instructions with bovine serum albumin as standard.

### Enzymatic assay

The method used for microsomal incubations is a modification of the method to measure 7-ethoxyresorufin hydroxylation by Zamaratskaia and Zlabek (2009). The incubation mixture in a final volume of 500 µl contained 0.25 mg microsomal protein (25 µl of 10 mg/ml protein), buffer (50 mM Tris, 5 mM MgCl2, pH 7.4) and a single probe substrate or substrate combination. The probe substrates assayed in this study were 7-ethoxyresorufin, coumarin, and BFC. The names and amounts of probe substrates and their respective metabolites are given in Table 1. The reactions were started by addition of 1 mM NADPH. Incubations were carried out for 7 min and then stopped by adding 20 µl of ice-cold 40% TCA.

**Table 1:** Names and amounts of probe substrates in microsomal incubations.

Isoform	Substrate	Metabolite	Ranges of substrate concentrations in the incubations, µM	Fluorescence detection excitation/emission
CYP1A	ER	resorufin	0.1–12.0	560/586 nm
CYP2A	coumarin	hydroxycoumarin	2.5–300.0	338/458 nm
CYP3A	BFC	HFC	0.4–120.0	410/538 nm

ER, 7-ethoxyresorufin; BFC, 7-benzyloxy-4-trifluoromethylcoumarin

HFC, 7-hydroxy-7-benzyloxy-trifluoromethylcoumarine

### Inhibition study

To investigate the ability of resorufin to inhibit coumarin and BFC metabolism, the incubations were performed in the presence of various concentrations of coumarin and BFC, and two concentrations of resorufin, 0.5 and 10 pmol. Control incubations without resorufin contained equivalent volumes of ethanol.

### Instrumentation and chromatographic separation

The HPLC system from Merck-Hitachi consisted of an on-line vacuum degasser (L-7612), pump (L-7100), autosampler (L-7200), fluorescence detector (L-7485), interface (D-7000) and D-7000 HPLC System Manager (v 4.1) software. The chromatographic separation of resorufin, HFC and 7-hydroxycoumarin in an injection volume of 5µl was achieved on a LiChrospher^®^ 100 RP-18 column (5 m, 250 mm × 4 mm), equipped with a guard column.

The isocratic elution was carried out with a mobile phase of a 50 mM phosphate buffer:methanol:acetonitrile (52:45:3, v/v) at a flow rate of 1.0 ml/min. The fluorescence detector was set at 560 and 586 nm for excitation and emission wavelengths to detect resorufin (0–3.3 min), the detection wavelengths were then switched to 338 and 458 nm to detect 7-hydroxycoumarin (3.3–5.0 min), and finally, to 410 and 538 nm to detect HFC (5.0–12 min).

### Kinetic analysis

Kinetic parameters (Michaelis-Menten constant Km, maximal velocity of the reaction Vmax and dissociation constant for inhibitor binding Ki) were calculated by fitting data to the Michaelis-Menten equation with GraphPad Prism version 4.0 for Windows, GraphPad Software (San Diego California, USA). Comparison of Km and Vmax values obtained in the incubations with a single substrate and with a substrate cocktail was performed using unpaired t-test with Welch's correction. Differences were regarded statistically significant when *p<*0.05.

## Results

The limit of quantitation (LOQ) of the method was determined as the lowest concentration that can be measured with acceptable precision and accuracy (below 15% variations) and was 2 pmol for resorufin and 7-hydroxycoumarin, and 10 pmol for 7-hydroxy-7-benzyloxy-triflouromethylcoumarine (HFC). Intra- and inter-assay variations did not exceed 15%.

**Figure 1:**
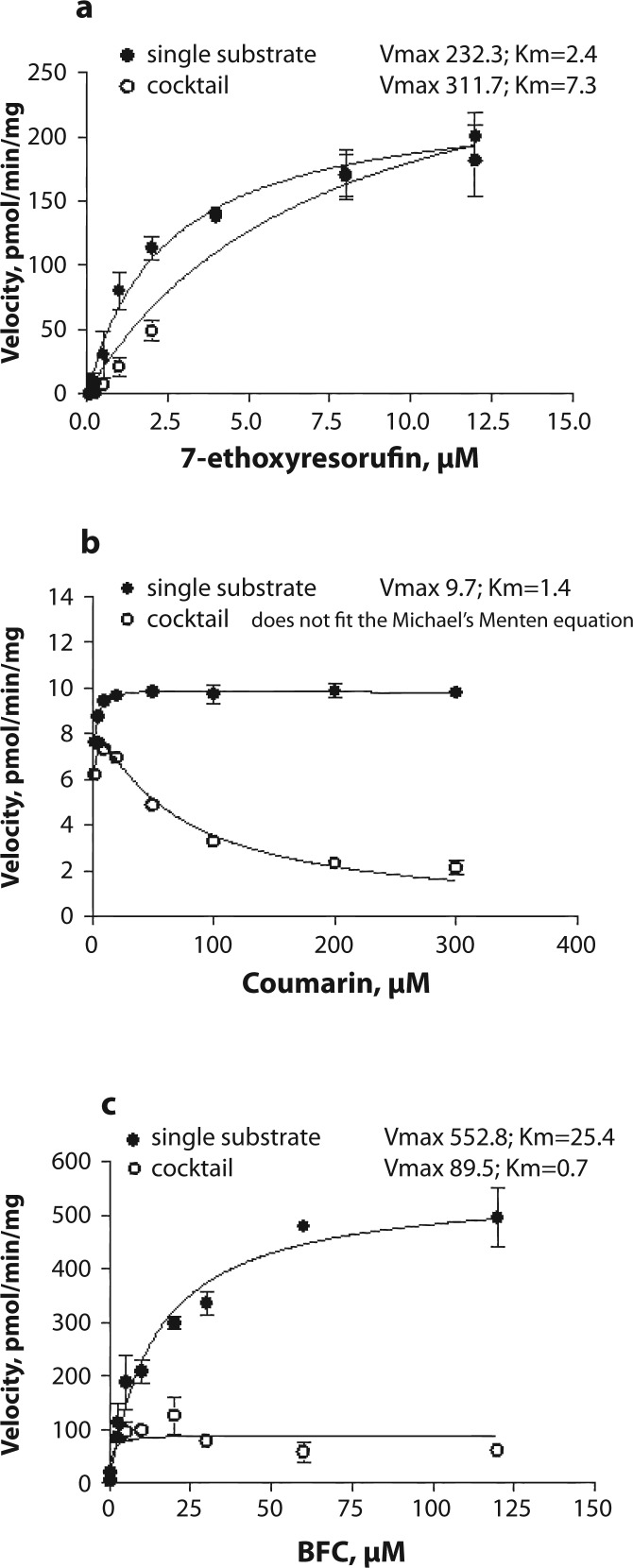
Saturation curves for a) 7-ethoxyresorufin deethylation (CYP1A), b) coumarin hydroxylation (CYP2A), and c) 7-benzyloxy-4-trifluoromethylcoumarin *O*-debenzylation (BFC; CYP3A) in porcine liver microsomes. Either a single probe substrate or the cocktail of three substrates was used. The activity was measured in two microsome pools from 5 pigs each at concentration of microsomal protein of 0.25 mg and various substrate concentrations.

**Figure 2:**
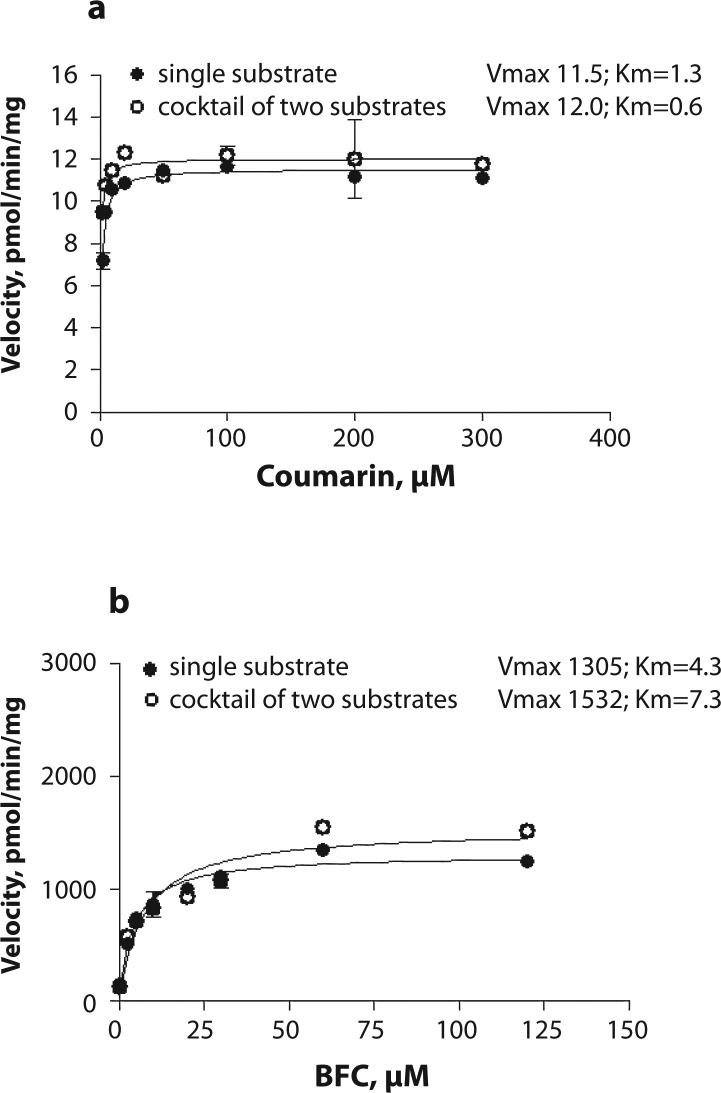
Saturation curves for a) coumarin hydroxylation (CYP2A) and b) 7-benzyloxy-4-trifluoromethylcoumarin *O-*debenzylation (BFC; CYP3A) in porcine liver microsomes. Either a single probe substrate or the cocktail of two substrates was used. The activity was measured in two microsome pools from 4 pigs each at the concentration of microsomal protein of 0.25 mg and various substrate concentrations.

Resorufin formation did not differ between the incubations with a single substrate or with a cocktail (Figure 1a). Vmax values were similar in both incubation sets (232.3 vs 311.7 pmol/min/mg, *p=*0.207). An increase in Km value from 2.4 to 7.3 was observed when using three substrates simultaneously but this increase was not statistically significant (*p=*0.103). Hydroxycoumarin formation from coumarin decreased when the substrate cocktail was used in the incubation mixture (Figure 1b). Vmax and Km values could not be compared because in the presence of the substrate cocktail 7-hydroxycoumarin formation did not follow the classical Michaelis-Menten kinetics. The rate of HFC formation from BFC decreased on using all three substrates in the incubation mixture (Figure 1c). Decreases in both Vmax from 552.8 to 89.5 pmol/min/mg and Km from 25.4 to 0.7 µM were observed (*p<*0.001 for both). Kinetic analysis revealed a non-competitive mode of inhibition of HFC formation by 7-ethoxyresorufin with Ki=1.1±0.15 (mean ± standard error). On excluding 7-ethoxyresorufin from the substrate cocktail inhibition of both hydroxycoumarin and HFC formation was eliminated (Figure 2a and 2b).

**Figure 3:**
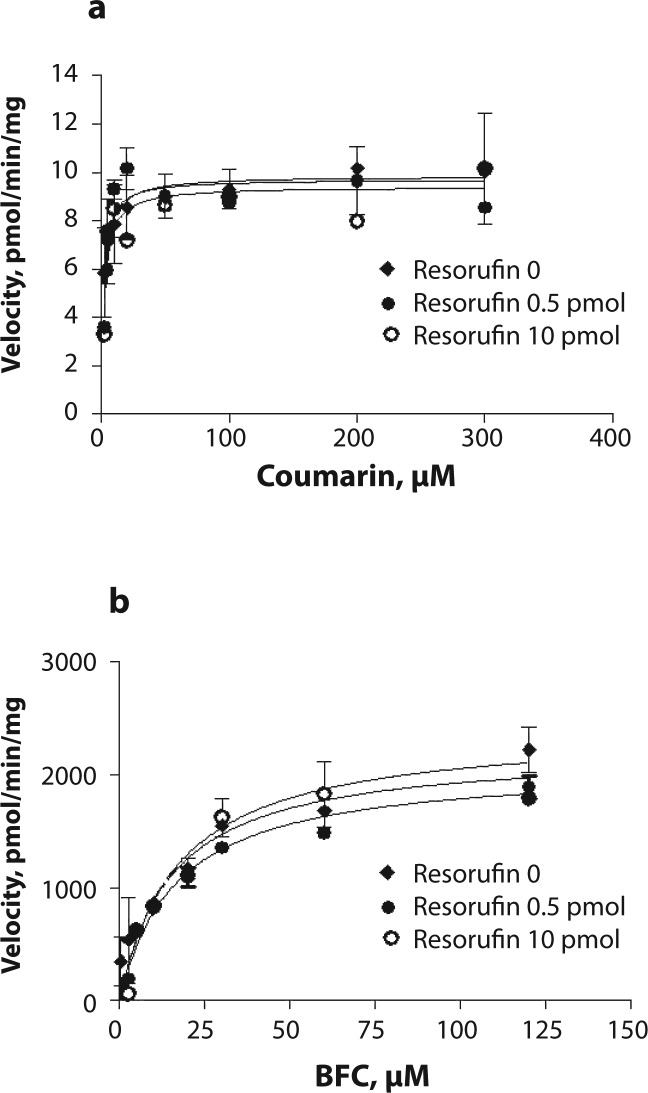
Saturation curve for a) coumarin hydroxylation (CYP2C) and b) 7-benzyloxy-4-trifluoromethylcoumarin *O-*debenzylation (BFC; CYP3A) in porcine liver microsomes in control incubations and in the presence of 0.5 and 10 pmol of resorufin. The activity was measured in two microsome pools from 4 pigs each at the concentration of microsomal protein of 0.25 mg and various substrate concentrations.

To further investigate the cause of inhibition, the incubations were performed in the presence of coumarin and BFC as substrates and two concentrations of resorufin, 0.5 and 10 pmol. Including resorufin in the incubations did not affect either coumarin hydroxylation or BFC *O*-debenzylation (Figure 3a and 3b).

## Discussion

A cocktail approach to measure the activity of several enzymes simultaneously is desirable because it can reduce both time and cost of analysis. However, the potential interference between substrates and their metabolites should be considered. Thus, the methods should be carefully validated before routine use. Often when validatating cocktail assays at simultaneous estimation of the activities of several CYPs, a single concentration of each substrate is used (He *et al.*, 2007). Only few studies included evaluation of the Km and Vmax values (Alden *et al.*, 2010). In the present study, we compared kinetic parameters obtained in the incubations with a single substrate with those obtained with a substrate cocktail. For this purpose, we used multiple concentrations of each substrate. We found that including all three substrates, 7-ethoxyresorufin, BFC and coumarin, in a single incubation mixture resulted in a strong inhibition of HFC formation from BFC and of hydroxycoumarin from coumarin. This inhibition was eliminated when 7-ethoxyresorufin was omitted from the incubation mixture. Given that the degree of inhibition increased with increasing concentration of the added 7-ethoxyresorufin, we hypothesized that 7-ethoxyresorufin itself or its metabolite resorufin acted as an inhibitor of metabolism of BFC to HFC, and of coumarin to hydroxycoumarin. To investigate which of these compounds exerts an inhibitory effect on BFC and coumarin metabolism, the incubations were performed in the presence of resorufin. Addition of resorufin alone did not affect BFC and coumarin metabolism suggesting that the inhibition observed in the presence of the cocktail was due to 7-ethoxyresorufin. Interestingly, Lahoz *et al.* (2008) used the cocktail approach to simultaneously assess the activities of 5 human isoforms in hepatocytes, where both 7-ethoxyresorufin and coumarin were included, and no inhibition of coumarin hydroxylation was observed. The disagreement between the study of Lahoz *et al.* (2008) and our present finding might be due to the use of different *in vitro* models and different concentrations of substrates in the incubations. Our results showed that at low concentrations of 7-ethoxyresorufin (up to 0.5 µM) no inhibition of coumarin hydroxylation occurred.

Further analysis revealed a non-competitive type of inhibition by 7-ethoxyresorufin of HFC formation from BFC. This type of inhibition, where the inhibitor interacts with the enzyme at a site other than the substrate binding site, was expected because all reactions studied were metabolized by different CYP450 isoforms. The results on the Ki value however should be interpreted with caution because 7-ethoxyresorufin is metabolized by microsomes and it was not possible to predict the exact the amount of 7-ethoxyresorufin in the incubations. To calculate Ki value more accurately, future analysis should be performed on a pure CYP3A isoform(s). The other limitation of the present method is that all CYP assays were performed under the same experimental conditions, whereas individual assays for each probe substrate are usually performed using optimized conditions for different enzymes; *e.g.* incubation buffer and amount of microsomal protein. This might also contribute to certain differences between the activities observed in the incubations with a single probe substrate or substrate cocktail.

## Conclusions

A simple and specific HPLC-based method with fluorescence detection was developed to separate resorufin, hydroxycoumarin and HFC. This assay may be useful for preliminary screening of CYP1A, CYP2A and CYP3A activities when low substrate concentrations are used. However, inhibition of CYP2A and CYP3A activities by 7-ethoxyresorufin does not allow accurate simultaneous kinetic analysis of the three isoforms studied. Thus the use of 7-ethoxyresorufin in substrate cocktails together with coumarin and BFC should be avoided.
